# LARS regulates cellular senescence and autophagy in liver cancer cells

**DOI:** 10.1038/s41419-026-09114-0

**Published:** 2026-07-17

**Authors:** Xiaodan Lin, Yanheng Chen, Caroline Hartmann, Lin-Feng Chen, Susan A. Martinis

**Affiliations:** 1 Department of Biochemistry, University of Illinois Urbana-Champaign, Urbana, IL USA; 2 Carl R. Woese Institute for Genomic Biology, University of Illinois Urbana-Champaign, Urbana, IL USA; 3 Cancer Center at Illinois, University of Illinois Urbana-Champaign, Urbana, IL USA; 4 Present Address: EnquBio, Inc., 11120 Roselle St Ste A, San Diego, CA USA

**Keywords:** Cell biology, Cancer

## Abstract

Human cytoplasmic leucyl-tRNA synthetase (LARS) is known to catalyze the ligation of leucine to tRNA^Leu^ during protein biosynthesis. However, LARS also acts as a nutrient sensor in a non-canonical activity to regulate cell growth. In this investigation, LARS was determined to be expressed at high levels in human liver cancer, which correlated with poor clinical outcomes in patients. Knockdown of LARS in HepG2 liver cancer cells suppressed cell proliferation and growth, yet promoted cell migration, without affecting global protein translation. Using data from RNA sequencing, differentially expressed genes in response to LARS knockdown were significantly clustered in the cellular senescence pathway. Elevated p21 and p16 expression with increased senescence-associated β-galactosidase activity was observed in LARS knockdown HepG2 cells. In addition, autophagy with increasing autophagic flux was triggered in LARS knockdown HepG2 cells. Furthermore, the depletion of LARS induced oxidative stress with increased production of reactive oxygen species and decreased mitochondrial membrane potential. Importantly, re-expression of shRNA-resistant LARS largely rescued the senescence, autophagy, and oxidative stress caused by LARS knockdown, confirming the specificity of these effects. These findings suggest that LARS plays a role in regulating liver cancer cell proliferation via multiple cellular responses and may serve as a promising therapeutic target in liver cancer.

## Introduction

Primary liver cancer is one of the most common cancer types worldwide and has only a 22% five-year survival rate based on U.S. cases diagnosed between 2013 and 2019 [1]. The American Cancer Society estimates that there will be 42,340 new patients and 30,980 deaths from liver cancer in the U.S. this year. Hepatocellular carcinoma (HCC) is the major form of liver cancer associated with several risk factors, including hepatitis B virus and hepatitis C virus infection, excessive alcohol intake, non-alcoholic steatohepatitis, diabetes, and smoking [2]. Clinical therapeutic treatments for HCC patients in the early-to-intermediate stage include surgical interventions, such as ablation, resection, or liver transplantation, and transarterial therapies. Advanced HCC patients rely on systemic therapies, as well as immunotherapy [2–4]. Identifying new biomarkers for liver cancer patients, especially in advanced stages, would provide an opportunity for enhanced clinical prediction, as well as facilitate current and new therapeutic strategies.

The aminoacyl-tRNA synthetases (aaRSs), a family of housekeeping enzymes best known for attaching amino acids to their cognate tRNAs in the first step of protein synthesis [5, 6], has been found to be embedded in a multitude of non-canonical activities including cell signaling, immune response, splicing, as well as transcriptional and translational regulation [7, 8]. As such, genetic variations and abnormal expression of aaRSs are associated with diverse human diseases and cancer types. Thus, we hypothesized that specific aaRSs might serve as potential biomarkers for disease diagnosis and therapeutic targets in tumorigenesis [9, 10].

In mammals, nine cytoplasmic aaRSs assemble with three aaRS-interacting multifunctional proteins (AIMPs 1-3) to form a multi-tRNA synthetase complex (MSC) [11, 12]. Human cytoplasmic leucyl-tRNA synthetase (LARS) is an essential member of the MSC for aminoacylation [13]. It also serves in a non-canonical role as a leucine sensor to mediate leucine signaling to mammalian target of rapamycin complex 1 (mTORC1) activation via RagD interaction and the Vps34-PLD1 pathway in parallel [14, 15]. Upon glucose deprivation, LARS undergoes post-translational modifications to block both mTORC1 activation and leucylation activity, thereby inhibiting protein translation [16, 17]. Subsequent to leucine starvation, LARS stability is promoted via interaction with prolyl-hydroxylase domain isoform 1 (PHD1) to further sustain mTORC1 activation and cell survival [18].

We investigated whether LARS is involved in cancer cell growth and tumorigenesis. Our analyses revealed that LARS expression is elevated in liver cancer patients and that depletion of LARS suppresses proliferation and growth of HepG2 liver cancer cells by inducing cellular senescence, autophagy, and oxidative stress. Collectively, these findings indicate that LARS knockdown activates multiple tumor-suppressive pathways, supporting LARS as a potential therapeutic target in liver cancer.

## Results

### LARS is overexpressed in HCC and correlates with poor clinical outcomes in cancer patients

**Fig. 1:**
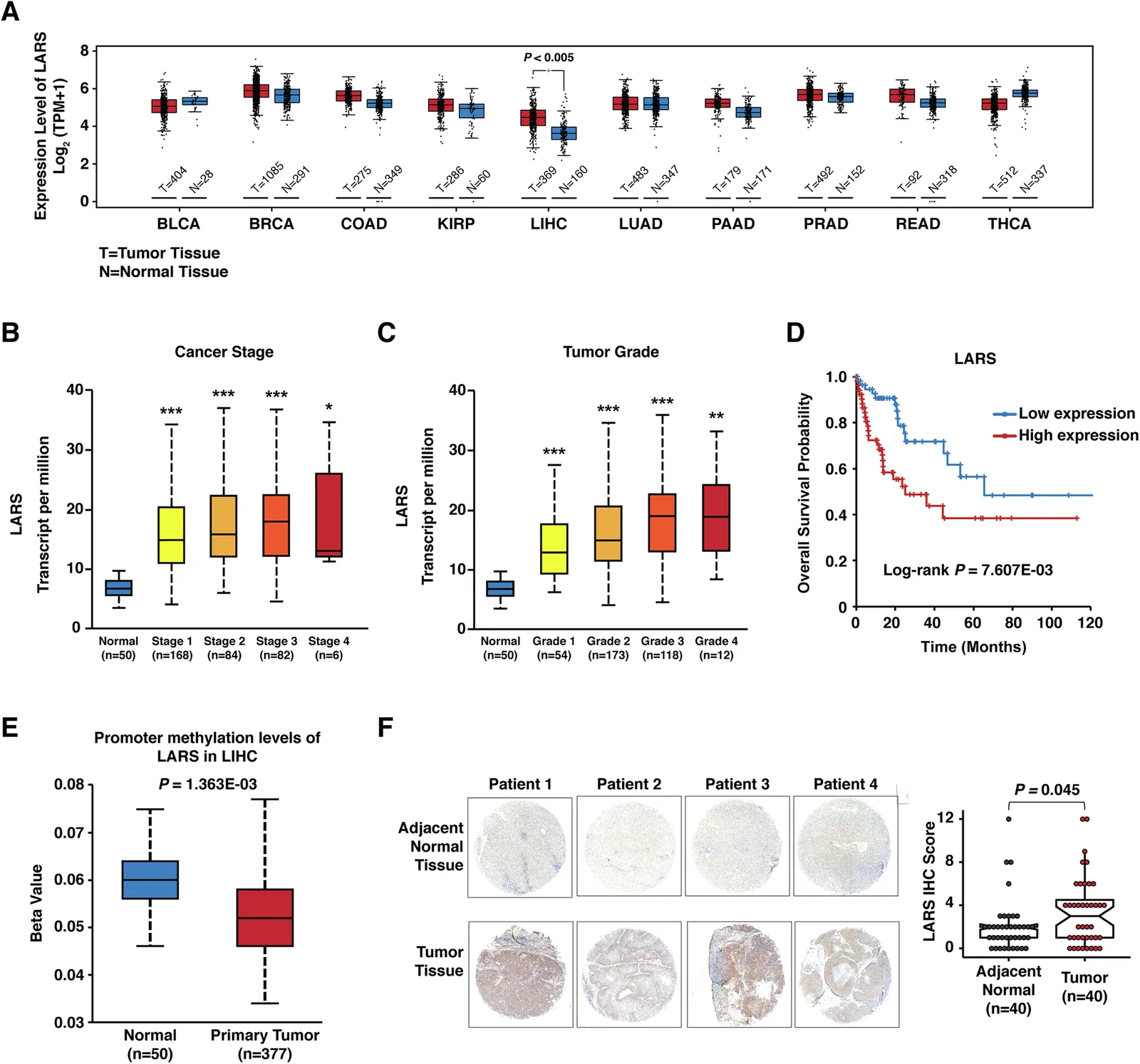
LARS expression is elevated in hepatocellular carcinoma and correlates with poor clinical outcomes. **A** Boxplot analysis showing LARS mRNA expression in normal liver tissues (N; blue) and tumor tissues (T; red) based on TCGA and GTEx database analysis. LARS expression levels across different pathological stages (**B**) and tumor grades (**C**) of HCC, as analyzed using the UALCAN database. **D** Kaplan-Meier overall survival analysis of HCC patients stratified by high (red) or low (blue) LARS expression, generated using TCGA data obtained from cBioPortal. **E** Comparison of LARS promoter methylation levels between normal liver tissues (*n* = 50; blue) and HCC tumor tissues (*n* = 377; red) based on UALCAN analysis. **F** Left: Representative immunohistochemical (IHC) staining images showing LARS protein expression in HCC tumors and paired adjacent normal liver tissues. Right: Quantitative scatterplot of LARS IHC staining scores from 40 paired HCC and adjacent normal liver samples. Abbreviations: TPM transcripts per million, BLCA bladder urothelial carcinoma, BRCA breast invasive carcinoma, COAD colon adenocarcinoma, KIRP kidney renal papillary cell carcinoma, LIHC liver hepatocellular carcinoma, LUAD lung adenocarcinoma, PAAD pancreatic adenocarcinoma, PRAD prostate adenocarcinoma, READ rectum adenocarcinoma, THCA thyroid carcinoma.

To explore the potential tumorigenic role of human cytoplasmic LARS, we first examined LARS transcriptional levels across a broad spectrum of human cancers using Gene Expression Profiling Interactive Analysis (GEPIA) [19], an online platform for gene expression analysis based on data from The Cancer Genome Atlas (TCGA) and the Genotype-Tissue Expression (GTEx) databases. We found that LARS expression was elevated in several cancer types, including breast invasive carcinoma, colon adenocarcinoma, pancreatic adenocarcinoma, and rectum adenocarcinoma. Notably, the most statistically significant upregulation of LARS was observed in liver hepatocellular carcinoma (LIHC) (Fig. 1A).

Given the marked difference in LARS expression between normal liver tissue and liver tumors, we focused on defining the potential role of LARS in liver cancer. LARS expression patterns across different tumor stages and grades were analyzed using the University of Alabama at Birmingham Cancer Data Analysis (UALCAN) portal [20], based on TCGA data. Notably, LARS mRNA levels were elevated at early stages and in lower-grade liver cancers, and this increase positively correlated with tumor development and progression (Fig. 1B, C). Kaplan-Meier survival analysis using TCGA data obtained from cBioPortal [21, 22] revealed that liver cancer patients with higher LARS expression exhibited significantly poorer overall survival (OS) (Fig. 1D). Furthermore, promoter methylation levels of the LARS gene were reduced in primary tumor tissues compared with normal liver tissue (Fig. 1E), suggesting that LARS overexpression in liver cancer may be driven, at least in part, by epigenetic transcriptional regulation.

We next examined LARS protein expression in 40 paired liver tumor samples and their corresponding adjacent normal tissues using immunohistochemical (IHC) staining. Consistent with the mRNA expression data, LARS protein levels were frequently elevated in tumor tissues compared with matched normal tissues (Fig. 1F). Taken together, these findings indicate that LARS is upregulated at both the transcript and protein levels and suggest that LARS may play a role in the development of liver cancer.

### Knockdown of LARS inhibits HepG2 liver cancer cell growth, but promotes cell migration

**Fig. 2:**
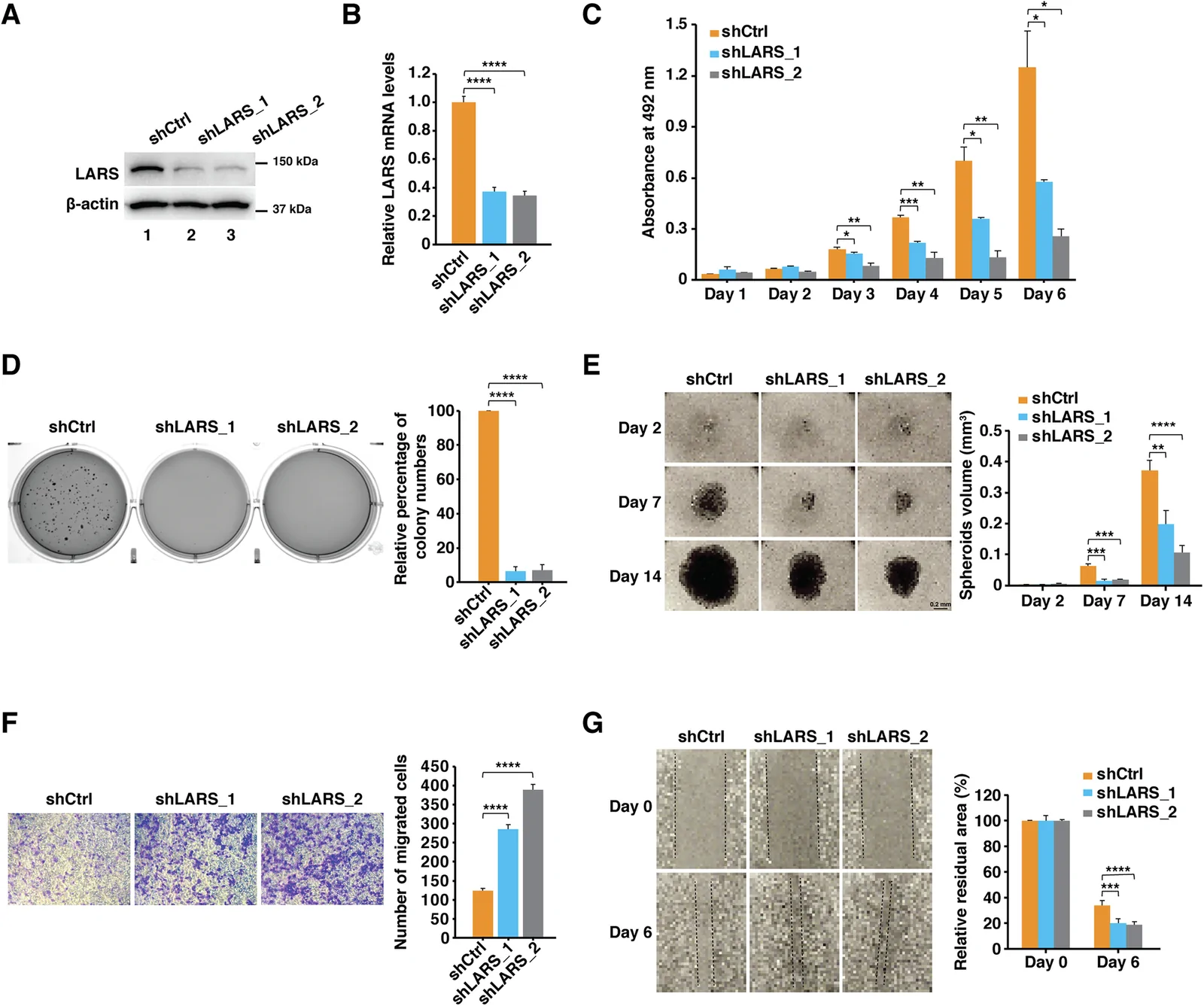
LARS depletion suppresses cell growth but enhances migratory capacity in HepG2 liver cancer cells. **A** Immunoblot analysis of LARS protein expression in control and LARS knockdown HepG2 cells. β-actin serves as the loading control. **B** Quantitative RT-PCR analysis of LARS mRNA levels in control and LARS knockdown HepG2 cells, normalized to GAPDH. **C** Cell growth of control and LARS knockdown HepG2 cells was assessed by MTS assay, with absorbance measured at 492 nm. **D** Representative images (left) and quantitative bar graph (right) of anchorage-independent growth assessed by soft agar colony formation assay in HepG2 cells. **E** Representative images (left) and quantitative bar graph (right) of 3D spheroid formation in control and LARS knockdown HepG2 cells at days 2, 7, and 14 (100X magnification; scale bar = 0.2 mm). **F** Representative images (left) and quantitative bar graph (right) of transwell migration assays performed in control and LARS knockdown HepG2 cells. **G** Representative images (left) and quantitative bar graph (right) of wound-healing migration assays at days 0 and 6. Dashed lines indicate the wound area (100X magnification).

To investigate the functional role of LARS in liver cancer, LARS expression was suppressed in human HepG2 liver cancer cells using shRNA-mediated lentiviral transduction. Two independent shRNAs targeting distinct regions of the LARS gene (shLARS_1 and shLARS_2) were employed. Western blot and qRT-PCR analyses confirmed efficient knockdown of LARS at both the protein and mRNA levels (Fig. 2A, B).

We next assessed the impact of LARS depletion on liver cancer cell growth. MTS assays revealed that LARS knockdown markedly inhibited HepG2 cell proliferation over a six-day period (Fig. 2C). Consistently, anchorage-independent growth was significantly reduced, as determined by soft agar colony formation assays (Fig. 2D). Furthermore, three-dimensional (3D) spheroid assays demonstrated that depletion of LARS significantly decreased spheroid volume in HepG2 cells expressing either of the shLARS constructs compared with shCtrl-expressing cells (Fig. 2E).

To determine whether LARS influences the migratory capacity of liver cancer cells, we performed transwell and wound healing assays. Unexpectedly, transwell migration assays showed a significant increase in the number of cells migrating through the membrane after 24 h in both LARS knockdown groups relative to control cells (Fig. 2F). Consistent with these findings, wound healing assays revealed accelerated wound closure in LARS-depleted HepG2 cells, indicating enhanced migratory ability (Fig. 2G). Collectively, these data indicate that LARS depletion suppresses HepG2 cell growth in both two- and three-dimensional culture systems while paradoxically promoting cell migration.

Reduced cancer cell proliferation coupled with enhanced migratory capacity has been associated with activation of epithelial-to-mesenchymal transition (EMT) during tumor progression [23, 24]. To determine whether EMT contributes to the increased migration observed following LARS knockdown, we examined the expression of key EMT markers. The levels of the mesenchymal marker N-cadherin and the epithelial marker E-cadherin were only modestly altered in LARS-depleted HepG2 cells (Fig. S2). Moreover, other mesenchymal markers, including Snail and Vimentin, were not upregulated upon LARS knockdown (Fig. S2). These results suggest that the enhanced migration observed in LARS-depleted HepG2 cells is not mediated by EMT activation.

### LARS depletion does not significantly affect global protein translation or mTORC1 signaling in HepG2 liver cancer cells

Given the canonical role of LARS in aminoacylation and protein synthesis [5, 7], we hypothesized that the reduced proliferation and growth observed in LARS-depleted HepG2 cells might result from impaired global protein translation. To test this, we employed bioorthogonal non-canonical amino acid tagging (BONCAT), in which the methionine analog azidohomoalanine (AHA) is incorporated into nascent polypeptides [25]. Following azide-alkyne click chemistry [26], newly synthesized AHA-labeled proteins were resolved by SDS-PAGE and detected by Western blotting (Fig. S1).

**Fig. 3:**
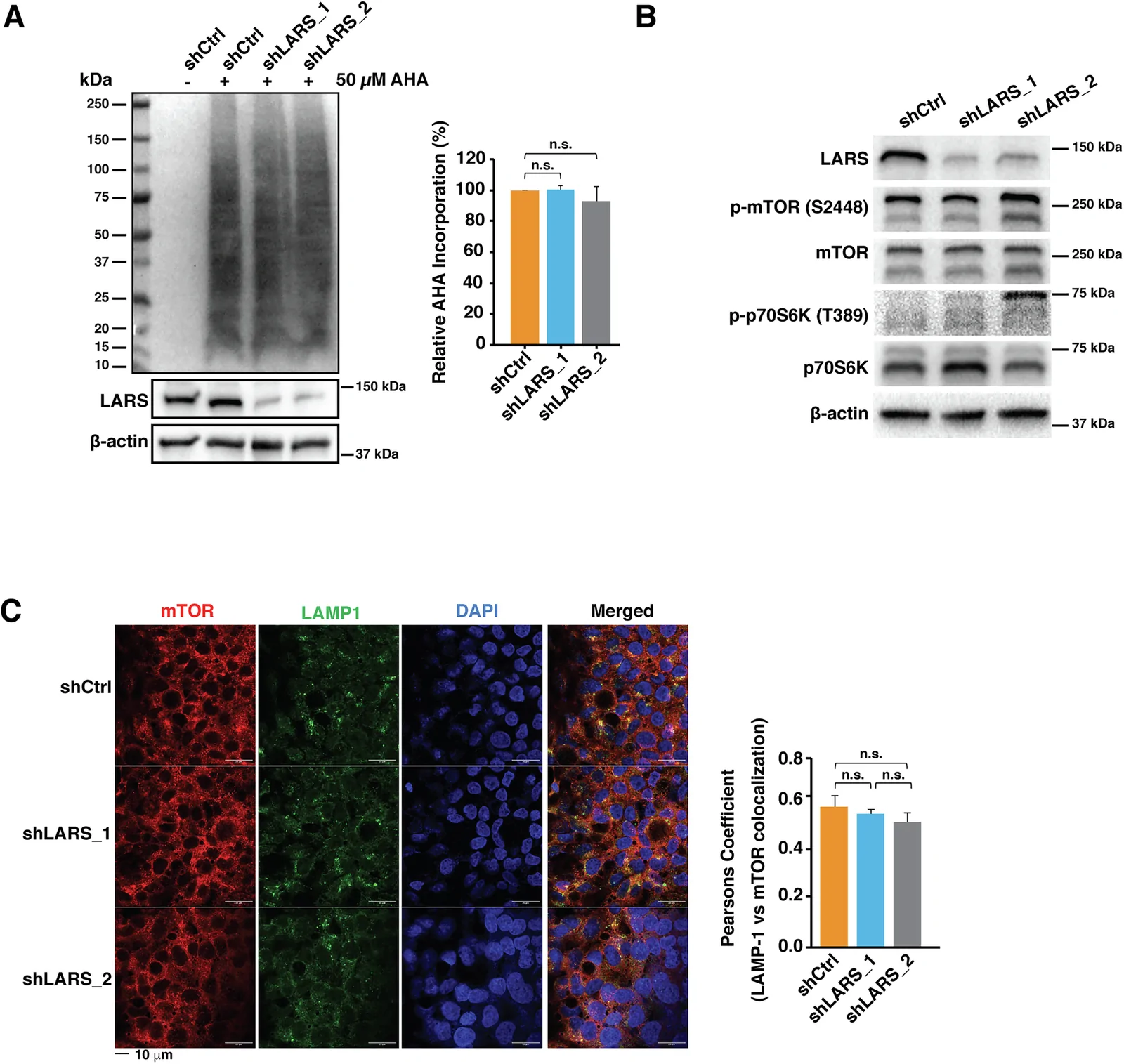
LARS depletion does not significantly affect global protein synthesis or mTORC1 signaling in HepG2 liver cancer cells. **A** Left: BONCAT-Western blot analysis of azidohomoalanine (AHA)-labeled newly synthesized proteins in control and LARS knockdown HepG2 cells. Right: Quantitative bar graph of newly synthesized protein levels. **B** Immunoblot analysis of LARS, mTOR, phospho-mTOR (Ser2448), p70S6K, and phospho-p70S6K (Thr389) protein levels in control and LARS knockdown HepG2 cells. β-actin serves as the loading control. **C** Left: Representative confocal microscopy images showing co-localization of the lysosomal marker LAMP1 (green) and mTOR (red) in control and LARS-knockdown HepG2 cells. Nuclei were counterstained with DAPI (blue). Right: Quantification of LAMP1-mTOR co-localization by Pearson’s correlation coefficient analysis.

Quantitative analysis revealed comparable levels of AHA incorporation between control and LARS knockdown HepG2 cells, indicating that LARS depletion does not significantly impair global protein synthesis (Fig. 3A). These findings suggest that the residual LARS remaining after shRNA-mediated knockdown (Fig. 2A, B) is sufficient to sustain overall translational capacity and that the observed defects in cell proliferation and growth likely arise from translation-independent mechanisms. Consistent with this interpretation, previous studies have demonstrated that partial depletion of aaRSs by siRNA or shRNA does not necessarily compromise global protein synthesis under basal culture conditions. For example, MARS knockdown does not affect global translation in vitro or in vivo unless cells are subjected to methionine deprivation [27]. Similarly, knockdown of IARS, EPRS, or LARS in C2C12 myoblasts does not alter protein synthesis rates, although LARS depletion enhances differentiation under serum withdrawal conditions [28].

In addition to its role in translation, LARS has been identified as an intracellular leucine sensor that regulates activation of the mTORC1 signaling pathway [14, 15], a central regulator of cell growth and metabolism frequently dysregulated in cancer. However, Western blot analysis revealed no significant changes in mTORC1 activity following LARS knockdown in HepG2 cells, as assessed by phosphorylation of mTOR and its downstream effector ribosomal S6 kinase (S6K1/p70S6K) (Fig. 3B). The activation of mTORC1 relies on its recruitment to the lysosomal surface [29]. To further investigate whether LARS depletion affects mTORC1 lysosomal localization and activation, we performed immunofluorescence co-staining analysis of mTOR and the lysosomal marker LAMP1 in control and LARS-knockdown HepG2 cells (Fig. 3C). Quantitative analysis of mTOR-LAMP1 colocalization revealed no significant difference between control and LARS-knockdown cells (Fig. 3C). Together, these data indicate that the reduced proliferation and growth of LARS-depleted HepG2 cells are unlikely to be mediated by impaired global protein translation or altered mTORC1 signaling.

### LARS knockdown remodels the transcriptomic landscape of HepG2 liver cancer cells

**Fig. 4:**
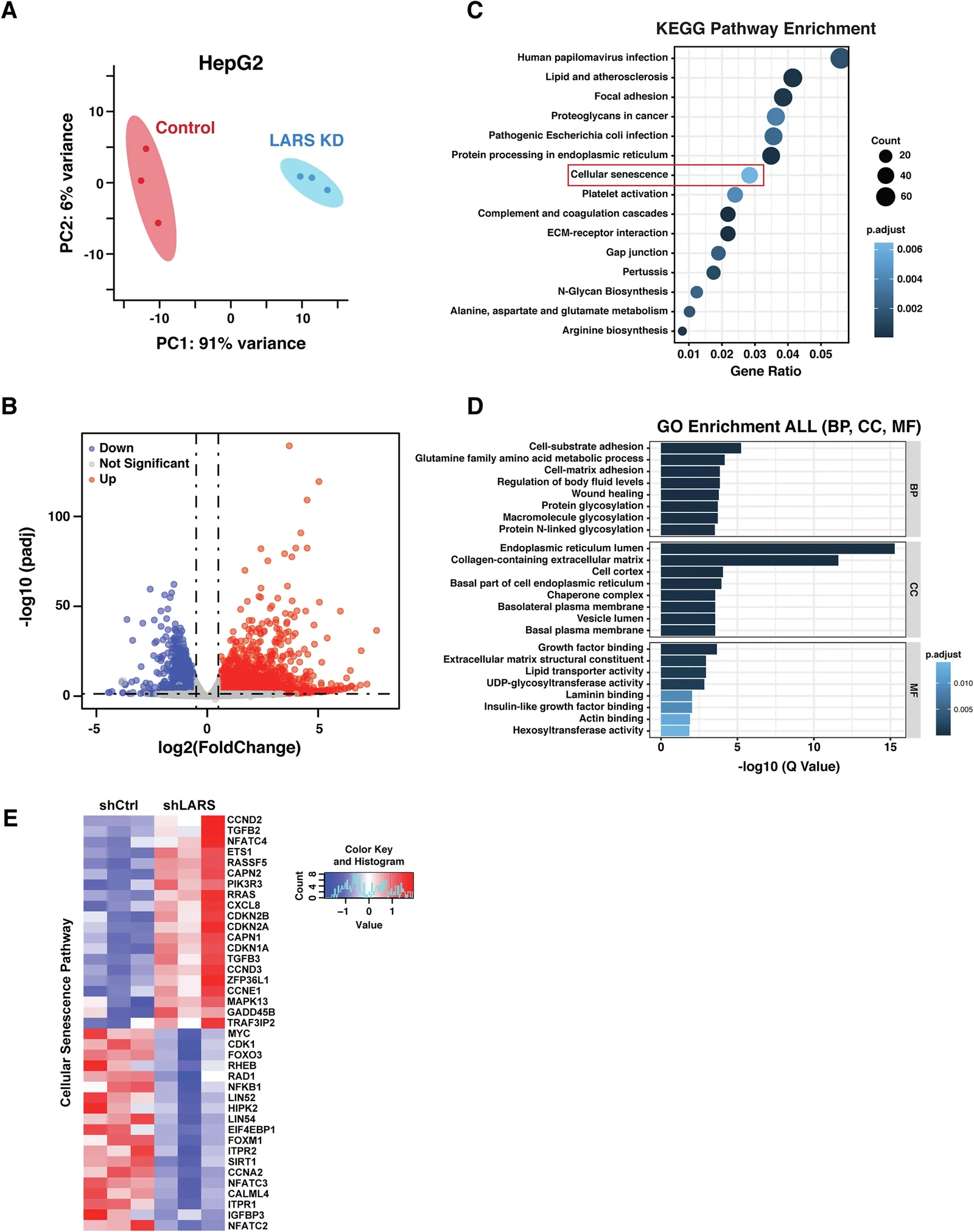
LARS depletion reshapes the transcriptomic landscape of HepG2 liver cancer cells. **A** Principal component analysis (PCA) of RNA-sequencing data from control and LARS knockdown HepG2 cells (*n* = 3 independent biological replicates per group). **B** Volcano plot showing differentially expressed genes following LARS knockdown. Red dots indicate upregulated genes, blue dots indicate downregulated genes, and gray dots indicate genes with no significant change ( | fold change | >1.5; FDR < 0.05). padj: adjusted *p*-values. **C** Top 15 enriched pathways identified by KEGG pathway analysis of significant differentially expressed genes. **D** Gene Ontology (GO) enrichment analysis of significantly altered genes, categorized into biological process (BP), cellular component (CC), and molecular function (MF). **E** Heatmap depicting differentially expressed genes associated with the cellular senescence pathway in HepG2 cells following LARS knockdown.

To elucidate the molecular mechanisms underlying the anti-proliferative effects of LARS depletion in HepG2 cells, we performed RNA sequencing (RNA-Seq) to analyze global transcriptional changes. Principal component analysis (PCA) revealed a clear separation between control and LARS knockdown samples, with principal component 1 (PC1) accounting for 91% of the variance and PC2 accounting for 6%, indicating substantial transcriptomic divergence between the two groups (Fig. 4A).

Differential expression analysis identified 3782 genes that were significantly altered upon LARS knockdown (fold change>1.5; false discovery rate [FDR] <0.05), as visualized by a volcano plot (Fig. 4B). Of these differentially expressed genes (DEGs), 2144 were upregulated and 1638 were downregulated in LARS-depleted HepG2 cells (Fig. 4B). Kyoto Encyclopedia of Genes and Genomes (KEGG) pathway analysis revealed that these DEGs were enriched in multiple pathways related to cancer cell proliferation and metabolism, including protein processing in the endoplasmic reticulum, cellular senescence, N-glycan biosynthesis, and alanine, aspartate, and glutamate metabolism (Fig. 4C). Consistently, Gene Ontology (GO) enrichment analysis across biological process (BP), cellular component (CC), and molecular function (MF) categories showed significant enrichment in pathways related to glutamine family amino acid metabolism, protein glycosylation, and UDP-glycosyltransferase activity (Fig. 4D).

Given that cellular senescence is a well-established mechanism for suppressing tumor cell proliferation [30], we further examined the expression patterns of senescence-associated genes. Heatmap analysis of DEGs involved in the cellular senescence pathway revealed upregulation of several key senescence markers, including the cell cycle inhibitors *CDKN1A*, *CDKN2A*, and *CDKN2B*, as well as the senescence-associated secretory phenotype (SASP) factor *CXCL8* (Fig. 4E). These transcriptional changes suggest activation of a senescence program in LARS-depleted HepG2 cells, providing a potential mechanistic basis for the observed growth inhibition.

### Knockdown of LARS induces cellular senescence in HepG2 liver cancer cells

**Fig. 5:**
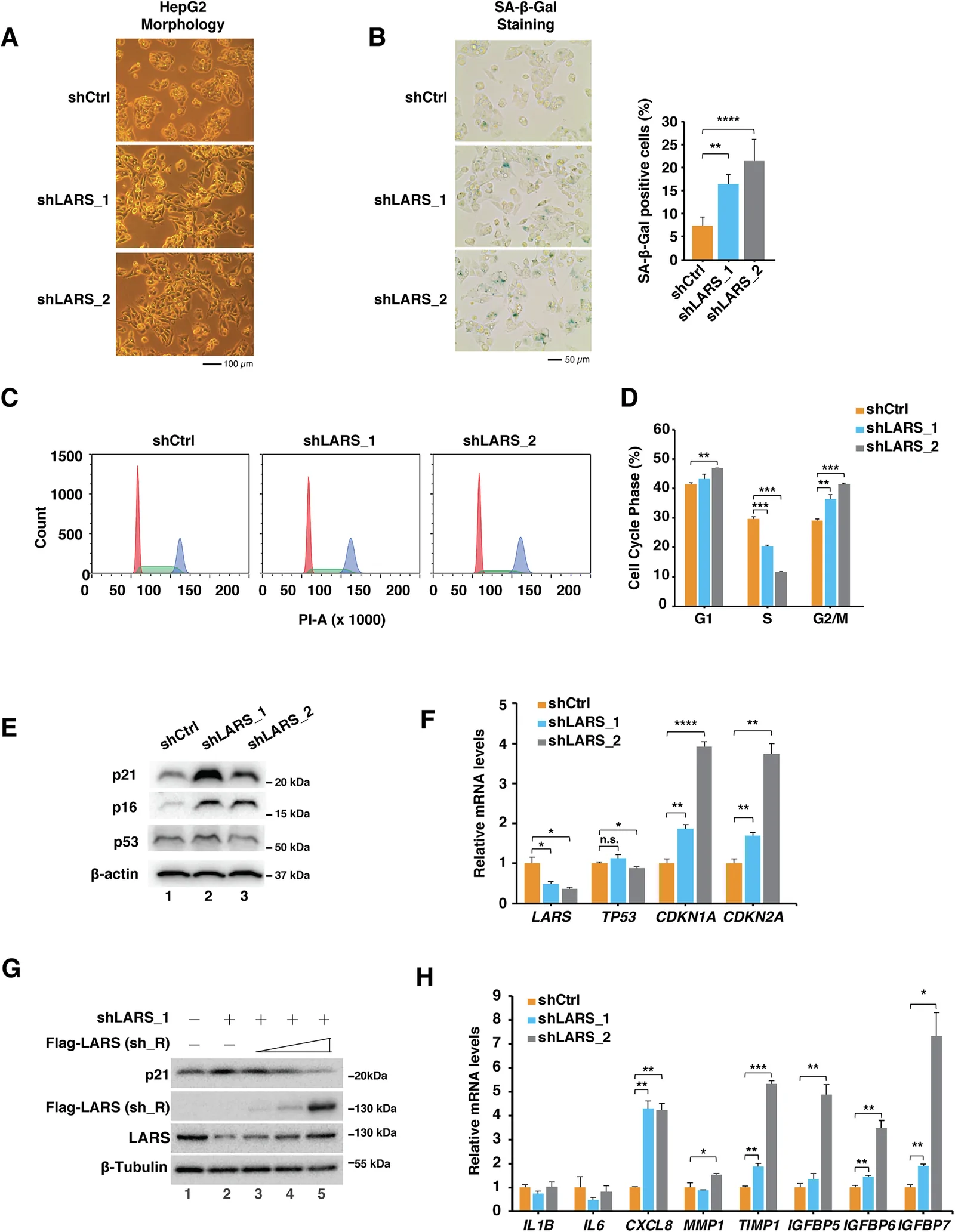
LARS knockdown induces cellular senescence in HepG2 liver cancer cells. **A** Representative phase-contrast images showing cellular morphology of control and LARS knockdown HepG2 cells (200X magnification; scale bar=100 µm). **B** Representative images of SA–β-Gal staining (left; scale bar=50 µm) and quantitative bar graph (right) in control and LARS knockdown HepG2 cells. **C** Flow cytometric analysis of cell-cycle distribution in control and LARS knockdown HepG2 cells stained with propidium iodide (PI). **D** Quantification of the percentage of cells in G1, S, and G2/M phases. **E** Immunoblot analysis of senescence-associated proteins p53, p21, and p16 in control and LARS knockdown HepG2 cells. β-actin serves as the loading control. **F** Quantitative RT-PCR analysis of LARS, TP53, CDKN1A, and CDKN2A mRNA levels, normalized to GAPDH, in control and LARS knockdown HepG2 cells. **G** Immunoblot analysis of the indicated proteins in control and LARS-knockdown HepG2 cells following re-expression of an shRNA-resistant Flag-tagged LARS1 construct. **H** Quantitative RT-PCR analysis of SASP gene expression in control and LARS knockdown HepG2 cells.

Since cellular senescence emerged as one of the most significantly enriched pathways in the KEGG analysis (*vide supra*), we hypothesized that senescence, a stable cell state characterized by irreversible growth arrest [31, 32], may contribute to the growth inhibition observed in LARS knockdown HepG2 cells. Compared with control cells, LARS-depleted HepG2 cells exhibited an enlarged and flattened morphology (Fig. 5A), a well-established phenotypic hallmark of senescent cells.

To further assess whether cellular senescence was induced by LARS knockdown, we examined senescence-associated β-galactosidase (SA-β-Gal) activity. Consistently, we observed a marked increase in SA-β-Gal–positive cells, with more than a 2- and 3-fold elevation in HepG2 cells expressing shLARS_1 and shLARS_2, respectively, compared to shCtrl-expressing cells (Fig. 5B).

Senescent cells are frequently associated with cell cycle arrest at the G1 and/or G2/M phases [33, 34]. Cell cycle analysis using propidium iodide (PI) staining revealed that LARS knockdown significantly reduced the proportion of cells in S phase, accompanied by an accumulation of cells in the G1 and G2/M phases (Fig. 5C, D). These findings suggest that the suppressed proliferation of LARS-depleted HepG2 cells is attributable to cell cycle blockade at the G1 and G2/M checkpoints.

Cell cycle arrest during senescence is typically mediated by activation of the p53-p21 and/or p16-pRB (retinoblastoma) pathways [30]. Accordingly, the cyclin-dependent kinase (CDK) inhibitors p21 and p16, encoded by *CDKN1A* and *CDKN2A*, respectively, are commonly upregulated in senescent cells to restrain cell cycle progression [35, 36]. In agreement with this model, LARS knockdown led to increased expression of p21 and p16 at both the mRNA and protein levels (Fig. 5E, F). Notably, the expression of the tumor suppressor p53 (*TP53*), a direct upstream transcriptional regulator of p21, was not significantly altered at either the mRNA or protein level, indicating a p53-independent mechanism of p21 induction.

To further verify the specificity of this phenotype, we performed rescue experiments using an shRNA-resistant LARS1 expression construct, designated as Flag-LARS(sh-R). Reintroduction of Flag-LARS(sh-R) into shLARS_1 knockdown cells markedly reduced p21 expression (Fig. 5G). Together, these findings demonstrate that the induction of p21 and the associated senescence phenotype are specifically caused by LARS depletion rather than off-target effects of shRNA-mediated knockdown.

In addition to cell cycle arrest, senescent cells develop a characteristic senescence-associated secretory phenotype (SASP), marked by elevated expression and secretion of inflammatory cytokines, chemokines, proteases, and growth factors that reinforce senescence through autocrine and paracrine signaling [37, 38]. Consistent with SASP activation, qRT-PCR analysis demonstrated that LARS knockdown significantly upregulated several SASP-associated genes, including *CXCL8*, *TIMP1*, *IGFBP5*, *IGFBP6*, and *IGFBP7* (Fig. 5H).

Programmed cell death via apoptosis is another major mechanism underlying tumor growth suppression [39]. However, key apoptotic effectors, including cleaved caspase-3 and poly(ADP-ribose) polymerase (PARP), were not increased in LARS-depleted HepG2 cells (Fig. S3). This observation aligns with previous reports indicating that senescent cells exhibit resistance to apoptosis [40–42]. Collectively, these findings demonstrate that LARS depletion suppresses HepG2 liver cancer cell proliferation primarily through induction of cellular senescence, rather than activation of apoptosis.

### LARS knockdown enhances autophagic flux in HepG2 liver cancer cells

**Fig. 6:**
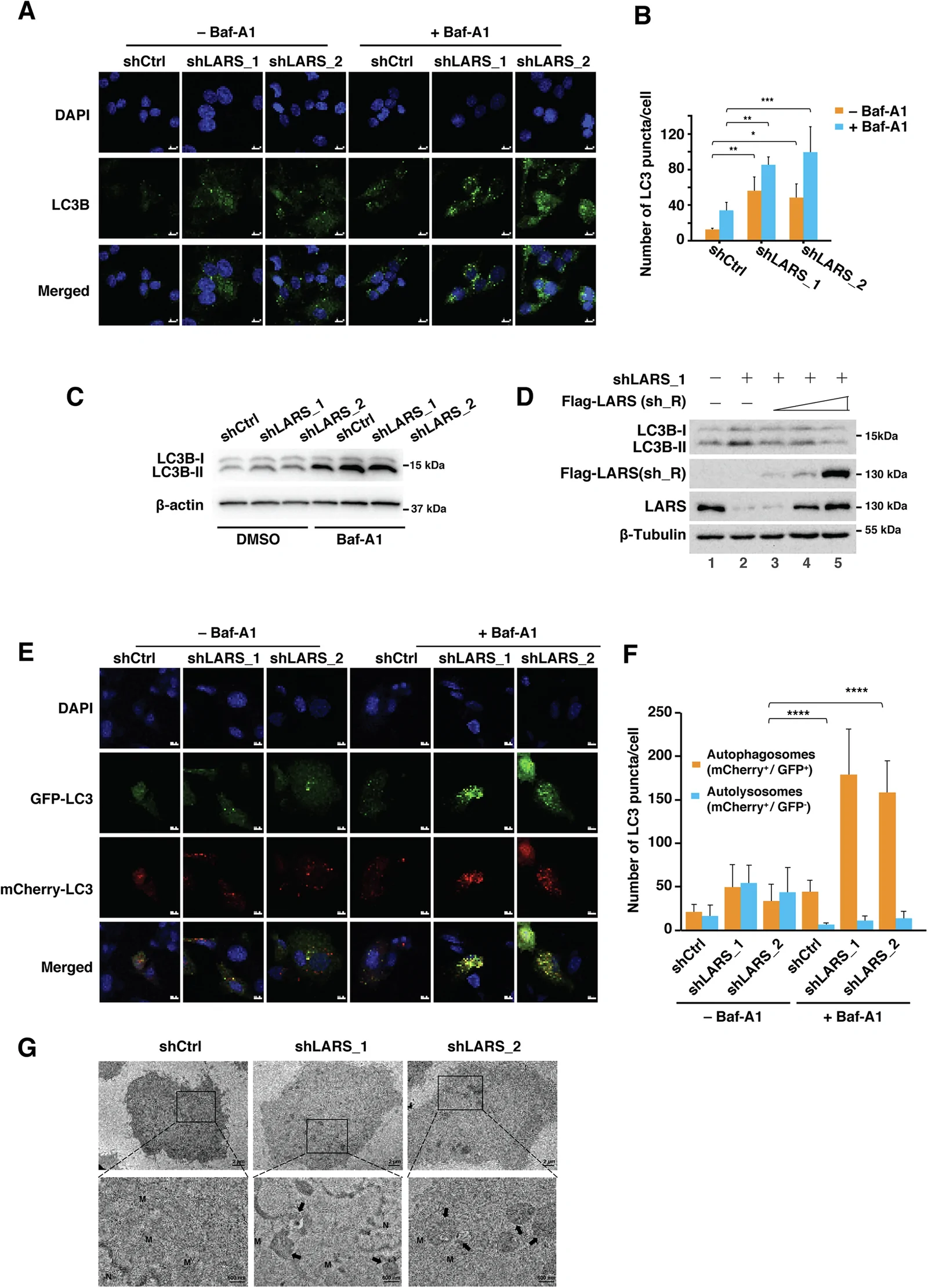
LARS knockdown enhances autophagic flux in HepG2 liver cancer cells. **A** Representative immunofluorescence images showing endogenous LC3B puncta (green) in control and LARS knockdown HepG2 cells, with nuclei counterstained with DAPI (blue), in the absence (left) or presence (right) of 25 nM bafilomycin A1 (Baf-A1). Scale bars=10 µm. **B** Quantification of the average number of LC3B puncta per cell, analyzed using ImageJ ( ≥ 30 cells per condition). **C** Immunoblot analysis of LC3B in control and LARS knockdown HepG2 cells in the absence (left) or presence (right) of 25 nM Baf-A1. β-actin serves as the loading control. **D** Immunoblot analysis of the indicated proteins in control and LARS-knockdown HepG2 cells following re-expression of an shRNA-resistant Flag-tagged LARS1 construct. **E** Representative confocal images of mCherry-GFP-LC3 reporter puncta in control and LARS knockdown HepG2 cells, with nuclei counterstained with DAPI (blue), in the absence (left) or presence (right) of 25 nM Baf-A1. Scale bars=10 µm. **F** Quantification of the average number of autophagosomes (yellow puncta) and autolysosomes (red puncta) per cell using ImageJ ( ≥ 10 cells per condition). **G** Representative transmission electron microscopy (TEM) images showing autophagosomes and autolysosomes in control and LARS knockdown HepG2 cells (N nucleus, M mitochondria, arrows indicate autophagosomes and autolysosomes). Scale bars: 2 µm (top) and 600 nm (bottom).

Autophagy is a critical tumor-suppressive mechanism that regulates cellular homeostasis and stress responses [43]. To determine whether autophagy contributes to the growth suppression observed in LARS-depleted HepG2 cells, we first examined the expression and localization of endogenous LC3B, a core marker of autophagy, by immunofluorescence staining. Compared with control cells, LARS knockdown cells exhibited a significant increase in LC3B-positive puncta (Fig. 6A, B), indicative of enhanced autophagosome formation.

Accumulation of LC3B can result from either increased autophagic flux or from impaired autophagic degradation caused by blockade of autophagosome-lysosome fusion or defective lysosomal acidification [44]. To distinguish between these possibilities, we performed autophagic flux assays using bafilomycin A1, a specific inhibitor of lysosomal acidification that prevents autophagosome degradation and thus blocks autophagic flux [45, 46]. As expected, bafilomycin A1 treatment increased LC3B puncta accumulation in control cells relative to untreated cells (Fig. 6A, B). Notably, LC3B puncta were further elevated in LARS knockdown cells in the presence of bafilomycin A1 (Fig. 6A, B), suggesting that LARS depletion enhances autophagic flux rather than impairing autophagosome clearance.

Consistent with these findings, Western blot analysis revealed that LARS knockdown promoted the conversion of cytosolic LC3B-I to the membrane-associated LC3B-II form, another hallmark of autophagosome formation (Fig. 6C). LC3B-II levels were further increased upon bafilomycin A1 treatment in LARS-depleted cells (Fig. 6C), providing additional evidence that LARS knockdown stimulates autophagic flux in HepG2 cells. Importantly, re-expression of the shRNA-resistant LARS1 construct (Flag-LARS (sh_R)) in shLARS_1 knockdown cells markedly reduced LC3B-II levels (Fig. 6D), confirming that the induction of autophagy is specifically attributable to LARS depletion and not an off-target effect of shRNA-mediated knockdown.

To further validate these observations, we employed a tandem mCherry-GFP-LC3 reporter to directly monitor autophagic flux. Because GFP fluorescence is quenched in the acidic lysosomal environment, whereas mCherry fluorescence remains stable, this system allows discrimination between autophagosomes (yellow puncta; mCherry⁺GFP⁺) and autolysosomes (red puncta; mCherry⁺GFP⁻) [44]. Following transient transfection of HepG2 cells with the mCherry-GFP-LC3 construct and incubation for 48 h, LC3 fluorescence was analyzed by confocal microscopy (Fig. 6E, F). LARS knockdown markedly increased the number of red LC3 puncta, indicating enhanced formation of autolysosomes. Moreover, inhibition of lysosomal acidification with bafilomycin A1 led to pronounced accumulation of yellow puncta in LARS-depleted cells compared with control cells, further confirming that LARS knockdown promotes active autophagic flux in HepG2 liver cancer cells.

Finally, transmission electron microscopy (TEM) analysis revealed an increased number of autophagosomes and autolysosomes in LARS knockdown cells, frequently enclosing damaged mitochondria and other cellular organelles (Fig. 6G). These ultrastructural features provide additional morphological evidence supporting the induction of autophagy following LARS depletion in HepG2 liver cancer cells.

### Knockdown of LARS induces reactive oxygen species accumulation and reduces mitochondrial membrane potential

Cellular stress is tightly linked to the induction of senescence, activation of autophagy, and suppression of cancer cell growth [47]. To determine whether LARS knockdown elicits a DNA damage response (DDR) in HepG2 cells, we first examined phosphorylation of histone H2AX at Ser139 (γ-H2AX), a canonical marker of DNA double-strand breaks (DSBs) [48–50]. However, γ-H2AX was not detectable in LARS-depleted cells by Western blot analysis (Fig. S4), indicating that LARS knockdown does not activate the DDR pathway.

**Fig. 7:**
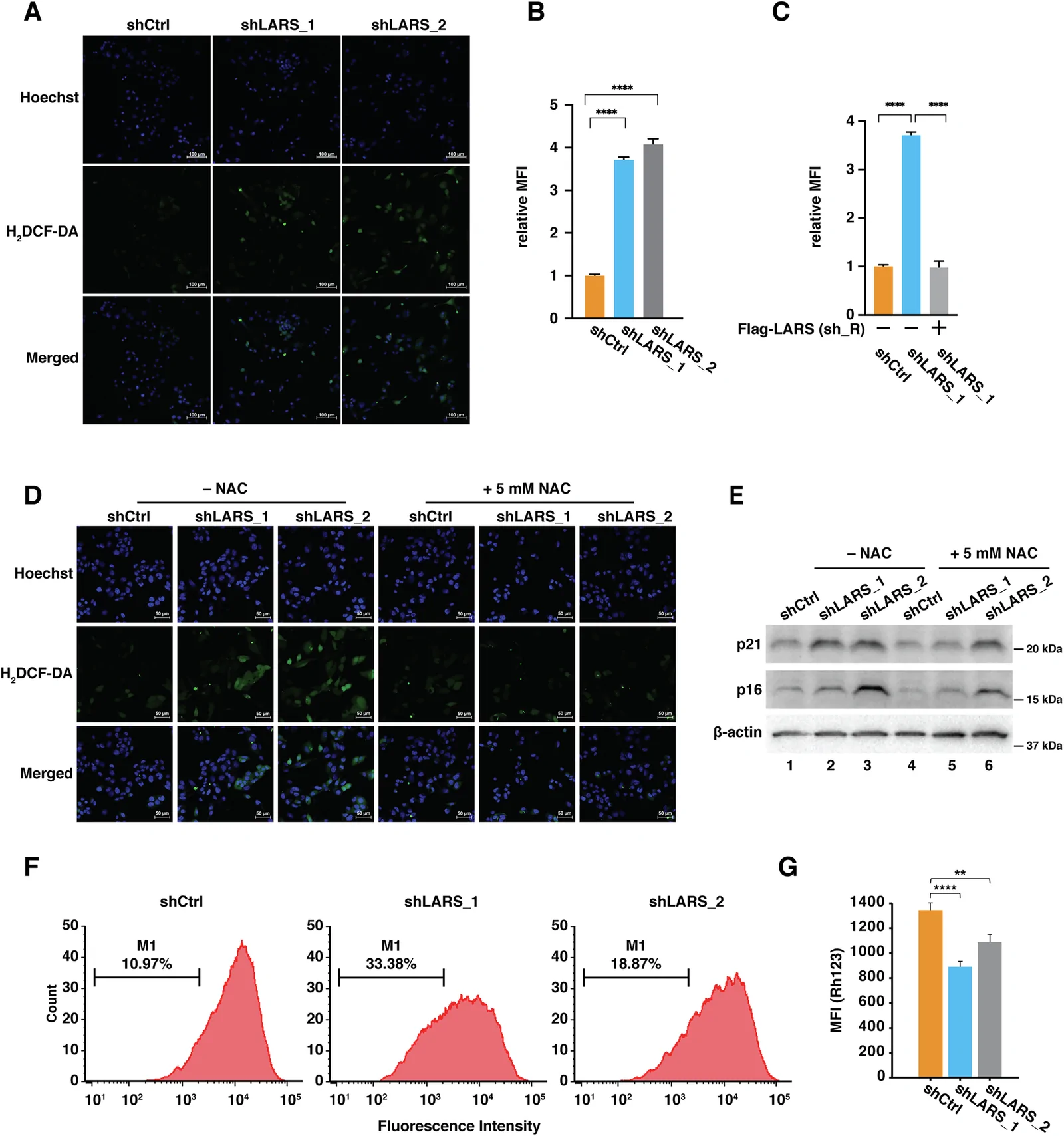
LARS knockdown induces cellular stress in HepG2 liver cancer cells. **A** Representative confocal microscopy images showing intracellular ROS levels in control and LARS knockdown HepG2 cells, detected by oxidation of H2DCF-DA to fluorescent DCF (green). Nuclei were counterstained with Hoechst 33342 (blue). Scale bars = 100 µm. **B** Quantification of intracellular ROS levels in control and LARS-knockdown HepG2 cells by flow cytometric analysis of DCF fluorescence. **C** Quantification of intracellular ROS levels in control and LARS-knockdown HepG2 cells following re-expression of an shRNA-resistant Flag-tagged LARS1 construct. ROS levels were determined by flow cytometric analysis of DCF fluorescence and are presented as median fluorescence intensity (MFI). **D** Representative confocal microscopy images showing intracellular ROS levels in control and LARS knockdown HepG2 cells in the absence and presence of NAC (5 mM) as in (**E**). **F** Flow cytometric analysis of mitochondrial membrane potential (MMP) in control and LARS knockdown HepG2 cells using Rhodamine 123 (Rh123). **G** Quantification of Rh123 median fluorescence intensity (MFI).

We next investigated whether LARS depletion induces oxidative stress in HepG2 cells by assessing intracellular reactive oxygen species (ROS) levels using the cell-permeable fluorescent probe 2′,7′-dichlorodihydrofluorescein diacetate (H₂DCF-DA). Confocal microscopy revealed a marked increase in ROS levels in LARS knockdown cells compared with control cells (Fig. 7A). This elevation in ROS was further confirmed by flow cytometric analysis (Fig. 7B). Importantly, re-expression of the shRNA-resistant LARS1 construct (Flag-LARS(sh_R)) in shLARS_1 knockdown cells markedly reduced ROS levels (Fig. 7C), demonstrating that the elevated oxidative stress is specifically attributable to LARS depletion rather than an off-target effect of shRNA-mediated knockdown.

Furthermore, treatment with the ROS scavenger N-acetyl-L-cysteine (NAC) significantly reduced ROS accumulation in LARS-depleted cells (Fig. 7D) and partially attenuated the upregulation of the senescence markers p21 and p16 (Fig. 7E), suggesting that oxidative stress contributes, at least in part, to the senescence phenotype induced by LARS knockdown.

Because elevated ROS levels are frequently associated with mitochondrial dysfunction [51], we next evaluated mitochondrial membrane potential using the fluorescent dye rhodamine 123 (Rh123). Flow cytometric analysis revealed that LARS knockdown significantly reduced mitochondrial membrane potential in HepG2 cells (Fig. 7F, G), indicative of impaired mitochondrial function. Collectively, these findings suggest that LARS depletion induces oxidative stress characterized by increased ROS generation and compromised mitochondrial membrane potential in HepG2 liver cancer cells, which may contribute to the observed senescence and autophagy phenotypes.

## Discussion

Previous studies have suggested that LARS may act as an oncogenic biomarker in certain cancers [52–54]. For example, LARS expression is upregulated in lung cancer and osteosarcoma, and its inhibition suppresses the growth of these cancer cells [52]. In contrast, a recent study reported a tumor-suppressive role for LARS in breast cancer, where LARS expression is downregulated, leading to decreased translation of tumor suppressors in a leucine codon-dependent manner [55]. Consistently, analysis of publicly available databases revealed lower LARS mRNA levels in bladder and thyroid cancers relative to normal tissues (Fig. 1A). These findings indicate that the role of LARS in cancer is likely context-dependent, acting either as a tumor-promoting or tumor-suppressive factor depending on cell type or tissue origin.

In this investigation, we identified a critical role for LARS in promoting liver cancer proliferation and growth. LARS expression was upregulated in liver tumor tissues and inversely correlated with clinical outcomes in patients (Fig. 1). Functionally, LARS depletion impaired HepG2 cell proliferation by inducing cellular senescence, enhancing autophagic flux, and triggering oxidative stress, without markedly affecting global protein translation. Importantly, re-expression of an shRNA-resistant LARS construct substantially reversed the induction of cellular senescence, autophagy, and ROS accumulation caused by LARS knockdown (Figs. 5G and 6D;7C), confirming that these phenotypes are directly attributable to loss of LARS. Together with the comparable effects observed using two independent LARS-targeting shRNAs, these findings support a functional role for LARS in maintaining mitochondrial function and preventing stress-induced senescence in hepatocellular carcinoma cells.

Recent studies have also highlighted emerging non-canonical roles of LARS1 in hepatobiliary malignancies. Shi et al. identified LARS1 as a prognostic biomarker and potential therapeutic target in hepatocellular carcinoma [56], whereas Liu et al. demonstrated that LARS1 promotes chemoresistance in intrahepatic cholangiocarcinoma through regulation of N-glycosylation [57]. Our findings complement and extend these observations by revealing that LARS depletion induces cellular senescence, oxidative stress, mitochondrial dysfunction, and autophagy in hepatocellular carcinoma cells. These results suggest that the oncogenic functions of LARS1 extend beyond amino acid sensing and protein translation and may involve broader regulation of cellular metabolism, stress responses, and tumor cell fitness.

An interesting observation in this study is that LARS depletion markedly inhibited cell proliferation despite having no detectable effect on global protein synthesis as measured by BONCAT (Figs. 2 and 3). This apparent paradox may reflect the temporal and functional separation between the canonical and non-canonical activities of LARS. BONCAT analysis was performed at a stage when overall translational capacity remained largely preserved, whereas the growth-inhibitory phenotype was associated with early induction of oxidative stress, mitochondrial dysfunction, autophagy, and cellular senescence. Increased ROS production and loss of mitochondrial membrane potential were observed following LARS knockdown and likely represent upstream events that activate p21/p16-dependent cell cycle arrest and senescence programs. Therefore, suppression of cell proliferation may occur independently of a measurable decline in bulk protein translation. In addition, LARS has been reported to function as a nutrient sensor that regulates signaling pathways involved in cell growth and metabolism [15, 16]. Consequently, depletion of LARS may impair cancer cell proliferation through disruption of these signaling and stress-response pathways rather than through a direct inhibition of global protein synthesis. Future studies examining the temporal sequence of ROS generation, senescence induction, and translational activity will help further define the mechanistic relationship among these events.

Cellular senescence is mediated in part by the cyclin-dependent kinase (CDK) inhibitors p21 and p16, which block the activity of distinct cyclin/CDK complexes and negatively regulate cell cycle progression [35, 58]. In HepG2 cells, LARS knockdown upregulated both p21 and p16, consistent with G1 and G2 cell cycle arrest (Fig. 5D, E). Notably, p53 levels were unaffected by LARS knockdown (Fig. 5E). It is possible that LARS depletion modulates post-translational modifications of p53, affecting its transcriptional activity [59], or alternatively, that p21 is regulated through transcriptional, post-transcriptional, or post-translational pathways independent of p53 [60]. As examples, transcription factors such as Sp1, E2Fs, STATs, and c-Myc can modulate p21 expression in a p53-independent manner [61].

Interestingly, although LARS knockdown reduced proliferation in both 2D and 3D growth models, it enhanced cell migration without clear induction of the EMT transition (Fig. 2). Senescent cells are known to secrete SASP factors that can promote migration and invasion [37]. Consistently, LARS depletion upregulated SASP genes such as *CXCL8* (encoding IL-8) and *IGFBP7* (Fig. 5H), both of which have been implicated in promoting cancer cell migration in breast, pancreatic, and glioma cells [62–64]. It is therefore possible that one or more SASP factors induced by LARS knockdown contribute to the enhanced migratory phenotype observed in senescent HepG2 cells.

Autophagy is a cytoprotective and tumor-suppressive mechanism activated in response to nutrient deprivation or other stress signals [43, 65], and it can interact with senescence in certain contexts [66, 67]. In oncogene-induced senescence, autophagy contributes to maintaining the senescence phenotype by supporting the production of SASP factors, involving TOR-autophagy spatial coupling compartment (TASCC) formation and mTOR activation through amino acids recycled from autophagy [66, 68]. Surprisingly, mTOR activity was not significantly altered in LARS-knockdown cells, as evidenced by immunoblot analysis and the unchanged colocalization of mTOR with LAMP1 (Fig. 6), suggesting that the enhanced autophagy observed in these cells occurs through an mTOR-independent mechanism. Stress-responsive p38 MAP kinase signaling has also been implicated in promoting mitochondrial ROS generation, autophagy, and selective induction of senescence rather than apoptosis [69]. Significantly, we consistently observed enhanced autophagic flux and increased phosphorylation of p38 MAP kinase in LARS-depleted HepG2 cells (Fig. S5). These findings suggest that activation of the p38 MAPK pathway may contribute to the induction of autophagy and senescence following LARS depletion, potentially through a mechanism involving oxidative stress.

Senescence can be induced by a variety of cellular stresses, including DNA damage, oncogene activation, loss of tumor suppressors, oxidative stress, mitochondrial dysfunction, and telomere shortening [30, 70, 71]. Our data indicate that LARS knockdown induces oxidative stress rather than canonical DNA damage, as evidenced by increased ROS accumulation and decreased mitochondrial membrane potential (Fig. 7). Importantly, treatment with the ROS scavenger N-acetyl-L-cysteine (NAC) not only reduced ROS levels but also partially attenuated p21 and p16 induction (Fig. 7D, E), suggesting that oxidative stress contributes to the senescence phenotype. The observation that NAC only partially suppressed p21 and p16 upregulation further suggests that ROS-independent mechanisms also participate in LARS depletion-induced senescence. Notably, the upregulation of p16 may represent a novel LARS-dependent mechanism mediating stress-induced senescence and influencing liver cancer progression. Recent studies have identified intracellular nucleotide sensors, such as PI5P4Kβ, that couple cellular GTP levels to metabolic adaptation and stress responses [72]. However, targeted metabolomic analysis revealed no significant changes in intracellular GTP or other major nucleotide pools following LARS depletion (Fig. S6). Furthermore, PI5P4Kβ expression remained unchanged (Fig. S7). These findings argue against a major role for altered nucleotide sensing or nucleotide depletion in the observed phenotype. Future studies will be required to identify the additional pathways that cooperate with oxidative stress to promote cellular senescence following LARS depletion.

In summary, our investigation identifies a critical role for LARS in sustaining liver cancer cell proliferation and growth. Depletion of LARS suppresses HepG2 cell proliferation by activating multiple stress-responsive and tumor-suppressive pathways, including cellular senescence, autophagy, and oxidative stress, independent of its canonical role in protein synthesis. One limitation of the present study is that LARS depletion was achieved by partial shRNA-mediated knockdown rather than inducible genetic deletion. Because LARS is an essential component of the translational machinery, complete loss of LARS may result in profound defects in cell viability that obscure its non-canonical functions. Future studies using inducible or conditional genetic models will be important to define the threshold requirements for LARS and to determine whether the pathways identified here operate similarly under conditions of more complete LARS loss. Given that LARS functions as a nutrient sensor for both leucine and glucose [15, 16], its depletion in liver cancer cells may induce a nutrient deprivation-like state and metabolic reprogramming [73], thereby limiting cell proliferation and growth. Whether these anti-proliferative mechanisms are activated simultaneously or occur in a defined spatiotemporal sequence in liver cancer cells remains to be determined. Nevertheless, the elevated expression of LARS in liver tumors and its inverse correlation with patient survival underscore LARS as a potential prognostic marker and therapeutic target in liver cancer.

## Materials and methods

### Analysis of LARS expression in human cancer databases

The online server, GEPIA (http://gepia.cancer-pku.cn) {19], was adopted to compare gene expression levels of LARS between normal and tumor tissues across various types of cancer, based on TCGA and the GTEx databases. Gene expression was log2(TPM+1) transformed. The one-way ANOVA method was used for differential analysis. The UALCAN (https://ualcan.path.uab.edu) [20] portal was utilized to plot expression profiles of the LARS gene among different cancer stages and tumor grades of liver cancer, as well as to compare promoter methylation levels of the LARS gene between normal and tumor samples in liver cancer. The cBio Cancer Genomics portal (cBioPortal, https://www.cbioportal.org) was employed to analyze the TCGA liver hepatocellular carcinoma database and to visualize survival probability in liver cancer patients [21, 22]. A Kaplan–Meier survival curve was generated to compare OS in cancer patients expressing high and low levels of LARS.

### Immunohistochemistry staining (IHC)

An array for human liver tissue, including 40 pairs of hepatocellular carcinoma and normal adjacent liver tissue, was purchased from US Biomax (#LV801b). Immunohistochemical staining was carried out by the Histology Laboratory at UIUC (Urbana, Illinois). The tissue slide was then scanned by a Nanozoomer 2.0-HT Slide Scanner (Hamamatsu). Staining intensity was classified as 0 (no stain), 1 (weak stain), 2 (moderate stain), or 3 (strong stain). The staining percentage was designated as 1 ( < 25%), 2 (25–50%), 3 (51–75%), or 4 ( > 75%). The final staining score was calculated by the product of color intensity and positive cell percentage, which ranged from 0 to 12.

### Cell culture, generation of LARS knockdown cell lines, and cell treatment

Human liver cancer HepG2 cells provided by Dr. J.K. Kemper (UIUC) and human embryonic kidney HEK293T cells provided by Dr. D.J. Shapiro (UIUC) were cultured in Dulbecco’s modified Eagle’s medium (DMEM) containing 4.5 g/L glucose and 10% (v/v) fetal bovine serum (FBS, #F0926, Sigma-Aldrich) at 37 ℃ in a humidified incubator with 5% CO_2_.

LARS knockdown cell lines were generated by lentiviral transduction using plasmids provided by Dr. J. Chen (UIUC). Scrambled shRNA as well as shRNA clones utilized for knocking down human LARS have been previously described [14]. Briefly, VSV-G and dR8.2 packaging plasmids, together with shRNA cloned in pLKO.1-puro lentiviral vector was used to co-transfect HEK293T cells at 80% confluence, using Lipofectamine 2000 (#11668-019; Invitrogen). The medium was changed after 24 h, and the medium containing lentivirus was harvested 48 h subsequent to transfection. The supernatant was harvested by centrifugation at 200 X *g* for 5 min to remove cell debris and then passed through 0.45 µm filter prior to use. HepG2 cells were transduced with lentiviral medium in the presence of 8 μg/mL of Polybrene. Total protein and RNA samples were then extracted after 48 h of selection using 2 μg/mL of puromycin. The efficiency of LARS knockdown was validated by Western blot and quantitative real-time PCR (qRT-PCR) assays.

For the *N*-acetyl-L-cysteine (#A9165, Sigma-Aldrich) inhibition assays, 5 mM was used to treat cells for 48 h. For bleomycin (provided by Dr. S. Blanke Laboratory at UIUC) studies, concentrations of 5 µg/mL and 10 µg/mL were used to treat HepG2 cells for 24 h to induce DNA damage.

### Generation of shRNA-resistant Flag-LARS and rescue of LARS knockdown

To generate an shLARS_1-resistant construct, silent mutations were introduced into the shLARS_1 target region (5′-CCTCACTTTGACCCAAGCTAT-3′) of pcDNA3.0-Flag-LARS by site-directed mutagenesis using the following primers: forward 5′-CCACAGGCAACTTCCTCACCCTTACCCAAGCTA-3′ and reverse 5′-GAAAATTTGTCAATAGCTTGGGTAAGGGTGAGGAAGT-3′. Mutagenesis PCR was performed with PrimeSTAR Max DNA Polymerase (Takara Bio, Cat. #R045A) according to the manufacturer’s instructions, and the introduced mutations were confirmed by Sanger sequencing.

For rescue experiments, HepG2 cells were depleted of endogenous LARS by lentiviral transduction of shLARS_1. Following knockdown, cells were transfected with the shLARS_1-resistant Flag-LARS construct using Lipofectamine 3000 (Thermo Fisher Scientific #L3000015) according to the manufacturer’s protocol. Forty-eight h post-transfection, cells were harvested for the indicated assays.

### Protein extraction and Western blot (WB) assay

Cells were harvested and lysed in ice-cold radioimmunoprecipitation assay (RIPA) buffer (10 mM Tris-HCl pH 7.6, 150 mM NaCl, 1 mM EDTA, 1% Triton X-100, 0.1% sodium dodecyl sulfate (SDS), and 0.1% sodium deoxycholate) supplemented with 1X protease inhibitor cocktail (#11836170001; Roche) and 1X phosphatase inhibitor cocktail (#P5726 and #P0044; Sigma-Aldrich) according to the manufacturer’s instructions. Proteins were quantified using *DC* (detergent-compatible) Protein Assay (#5000116, Bio-Rad). Equal amounts (10–20 µg/well) of protein were separated via SDS-PAGE and then transferred to 0.2 µM Immun^®^-Blot PVDF membranes (#1620177, Bio-Rad). Membranes were immunoblotted with specific primary and secondary antibodies (Table 1). Protein bands were visualized using SuperSignal™ West Femto Maximum Sensitivity Substrate (ECL) (#34095; ThermoFisher) and detected by ChemiDoc Imaging System (Bio-Rad). Protein β-actin served as the loading control. Western blot experiments for each protein tested were repeated at least three times.

**Table 1:** Antibody source and use.

Antibodies	Source (catalog number)	Dilution	Assays
**Rabbit anti-human LARS**	Zhang & Martinis [78]	1:4000	WB
**Rabbit polyclonal anti-human LARS**	Sigma-Aldrich (HPA040881)	1:100	IHC
**Rabbit monoclonal anti-mTOR**	Cell Signaling Technology (2983)	1:1000	WB
**Rabbit polyclonal anti-phospho-mTOR**	Cell Signaling Technology (2971)	1:1000	WB
**Rabbit polyclonal anti-p70 S6 Kinase**	Cell Signaling Technology (9202)	1:1000	WB
**Rabbit polyclonal anti-phospho-p70 S6 Kinase**	Cell Signaling Technology (9205)	1:1000	WB
**Rabbit monoclonal anti-p21**^**Waf1/Cip1**^	Cell Signaling Technology (2947)	1:1000	WB
**Rabbit monoclonal anti-p16**	Cell Signaling Technology (80772)	1:1000	WB
**Rabbit monoclonal anti-LC3B**	Cell Signaling Technology (3868)	1:1000/1:500	WB/IF
**Mouse monoclonal anti-p53**	Santa Cruz (sc-126)	1:500	WB
**Rabbit polyclonal anti-phospho-p53 (Ser15)**	Cell Signaling Technology (9284)	1:1000	WB
**Rabbit polyclonal anti-acetyl-p53 (Lys382)**	Cell Signaling Technology (2525)	1:1000	WB
**Rabbit polyclonal anti-Caspase 3**	Cell Signaling Technology (9662)	1:1000	WB
**Rabbit monoclonal anti-PARP**	Cell Signaling Technology (9532)	1:1000	WB
**Rabbit monoclonal anti-E-Cadherin**	Cell Signaling Technology (3195)	1:1000	WB
**Rabbit monoclonal anti-N-Cadherin**	Cell Signaling Technology (13116)	1:1000	WB
**Rabbit monoclonal anti-Vimentin**	Cell Signaling Technology (5741)	1:1000	WB
**Rabbit monoclonal anti-Snail**	Cell Signaling Technology (3879)	1:1000	WB
**Rabbit monoclonal anti-phospho-Histone H2A.X (Ser139)**	Cell Signaling Technology (9718)	1:1000	WB
**Rabbit monoclonal anti-p38 MAPK**	Cell Signaling Technology (8690)	1:1000	WB
**Rabbit monoclonal anti-phospho-p38 MAPK**	Cell Signaling Technology (4511)	1:1000	WB
**Rabbit monoclonal anti-β-actin**	Cell Signaling Technology (4970)	1:1000	WB
**Beta Tubulin Polyclonal antibody**	Proteintech (10094-AP)	1:1000	WB
**Goat anti-rabbit IgG, peroxidase-labeled**	Sera Care (5220-0335)	1:10,000	WB
**Goat anti-mouse IgG, peroxidase-labeled**	Sera Care (5220-0341)	1:10,000	WB

### RNA isolation and quantitative real-time PCR (qRT-PCR) assay

Total RNA was isolated using an RNeasy Mini Kit (#74104, Qiagen) and cDNA was synthesized using iScript cDNA Synthesis Kit (#1708891, Bio-Rad) according to the manufacturers’ instructions. Then the qPCR reaction was conducted using iTaq Universal SYBR^®^ Green Supermix (#1725121, Bio-Rad), specific primers (Table 2), and a Real-Time PCR System (Applied Biosystems QuantStudio 3) to determine the expression of target genes. The mRNA levels of each gene were normalized to the housekeeping gene *GAPDH*.

**Table 2:** Sequences of primers for each gene tested in the qPCR assay.

Gene name	Forward primer (5’-3’)	Reverse primer (5’-3’)
***LARS***	TAC AAG AAC ATG CTT GCT CA	TCA ATC AGC CAC TGC TCA TC
***TP53*** ***CDKN1A***	GAG GTT GGC TCT GAC TGT ACC TGT CCG TCA GAA CCC ATG C	TCC GTC CCA GTA GAT TAC CAC AAA GTC GAA GTT CCA TCG CTC
***CDKN2A***	ATG GAG CCT TCG GCT GAC T	GTA ACT ATT CGG TGC GTT GGG
***IL1B***	TTC GAC ACA TGG GAT AAC GAG G	TTT TTG CTG TGA GTC CCG GAG
***IL6***	CCT GAA CCT TCC AAA GAT GGC	TTC ACC AGG CAA GTC TCC TCA
***CXCL8***	ACT GAG AGT GAT TGA GAG TGG AC	AAC CCT CTG CAC CCA GTT TTC
***MMP1***	AAA ATT ACA CGC CAG ATT TGC C	GGT GTG ACA TTA CTC CAG AGT TG
***TIMP1***	ACC ACC TTA TAC CAG CGT TAT GA	GGT GTA GAC GAA CCG GAT GTC
***IGFBP5***	TGA CCG CAA AGG ATT CTA CAA G	CGT CAA CGT ACT CCA TGC CT
***IGFBP6***	GAG GGG CTC AAA CAC TCT ACG	CCA TCC GAT CCA CAC ACC A
***IGFBP7***	ATC CCG ACA CCT GTC CTC AT	CCC AGC CAG TTA CTT CAT GCT
***GAPDH***	ACC ACC AAC TGC TTA GCA CC	CCA TCC ACA GTC TTC TGG GT

### Cell viability measurement (MTS assay)

HepG2 cells were transduced with the indicated lentiviruses and followed by puromycin selection for 48 h. Cells were then seeded into a 96-well plate with an initial density of 2000 cells/well in triplicate for cell viability determination via 3-(4,5-dimethylthiazol-2-yl)-5-(3-carboxymethoxyphenyl)-2-(4-sulfophenyl)-2H-tetrazolium (MTS) assay (#G5430, Promega) according to the manufacturer’s instructions. Absorbance was measured at 492 nm using a microplate reader (ThermoFisher).

### Anchorage-independent colony formation assay

A total of 5000 cells, resuspended in 2X DMEM, were seeded into 6-well plates containing a 0.6% Difco™ Noble agar base layer and a 0.35% Difco™ Noble agar top layer. Cells were cultured at 37 °C in a humidified incubator with 5% CO₂ for 3 weeks. Colonies were then stained with 0.01% crystal violet (#C3886, Sigma-Aldrich) and visualized using a ChemiDoc Imaging System (Bio-Rad). Formed colonies were counted manually.

### 3D spheroid formation assay

Cells were trypsinized and seeded at 100 cells per well in ultra-low attachment 96-well plates (#CLS4520, Corning). Cells were cultured at 37 °C in a humidified incubator with 5% CO₂ for 2 weeks. Culture medium was replaced every 2–3 days, and spheroid growth was imaged at indicated time points using an inverted microscope at 100X magnification (VWR).

### Transwell migration assay

Cell migration assays were performed using a 24-well Transwell apparatus containing 8-µm membrane inserts (#3422, Corning). Briefly, cells re-suspended in serum-free DMEM medium were seeded into the upper chamber. Then, DMEM supplemented with 10% FBS was added to the lower chamber as a chemoattractant. After incubation at 37 ℃ for 24 h, non-migrated cells on the upper surface of the membrane were removed using wet cotton swabs, whereas cells migrated to the lower surface were fixed with ice-cold methanol for 20 min and then stained with 0.2% crystal violet at room temperature for 20 min. Images were captured using inverted microscopy at 200X magnification (VWR).

### Wound healing migration assay

Cells were seeded into 6-well plates one day prior to the assay to achieve a confluent monolayer. A wound scratch was created using a 200 µL pipette tip. Cells were gently washed with 1X PBS to remove debris and then incubated in serum-free DMEM. Images of the wound were captured immediately (Day 0) using an inverted microscope at 100X magnification (VWR) to record initial wound width. Cells were maintained at 37 °C in a humidified incubator with 5% CO₂, and images were acquired every 24 h until Day 6, when the wound was nearly closed.

### BONCAT-AHA nascent protein incorporation assay

Following two days of puromycin selection, control and LARS knockdown HepG2 cells were seeded into 6-cm dishes and cultured to reach 80–90% confluency. Cells were gently washed twice with 1X PBS and incubated in L-methionine-free DMEM supplemented with 10% FBS for 30 min to deplete intracellular methionine. Cells were then labeled by incubation in L-methionine-free DMEM containing 10% FBS and 50 µM L-azidohomoalanine (AHA; #C10102, Invitrogen) for 2 h. After labeling, cells were washed twice with 1X PBS and lysed on ice for 30 min in lysis buffer containing 50 mM Tris-HCl (pH 8.0), 1% SDS, 1 mM phenylmethylsulfonyl fluoride (PMSF), 1X protease inhibitor cocktail, 1X phosphatase inhibitor cocktail, and 250 U/mL benzonase endonuclease. Lysates were clarified by centrifugation at 14,000 rpm for 5 min at 4 °C using a Microfuge® 18 centrifuge (Beckman Coulter), and supernatants were collected. Protein concentrations were determined using the DC Protein Assay (Bio-Rad). Equal amounts of protein were subjected to click chemistry with alkyne-tagged biotin using the Click-iT™ Protein Reaction Buffer Kit (#C10276, Invitrogen) according to the manufacturer’s instructions. Proteins were subsequently precipitated using methanol/chloroform/water extraction. The resulting protein pellets were air-dried and resuspended in 2X Laemmli sample buffer (#1610737, Bio-Rad), followed by incubation at 70 °C for 10 min. AHA-incorporated newly synthesized proteins were detected by Western blot using streptavidin-HRP (1:10,000 dilution) in Tris-buffered saline containing 0.1% Tween 20 and 1% BSA (#A7906, Sigma-Aldrich).

### RNA-Seq analysis

Total RNA samples were extracted using an RNeasy mini kit (Qiagen) from HepG2 control and LARS knockdown cells, with three biological replicates per group, and submitted to the Roy J. Carver Biotechnology Center at UIUC for RNA-Seq service. RNA quality was assessed on an AATI fragment analyzer before library preparation and construction. Deep sequencing was performed on an Illumina NovaSeq 6000 system with 100-base-pair (bp) single-read mode. Differentially expressed genes (DEGs) between the control and LARS knockdown groups were identified using the R package DESeq2 [74]. DEGs with a |fold change | > 1.5 and false discovery rate (FDR) < 0.05 are considered significant. Enrichment analysis for the Geno Ontology (GO) and KEGG pathways was conducted to annotate and cluster all DEGs in R using the clusterProfiler package [75, 76]. A *p*-value of less than 0.05 was set as the threshold for the GO and KEGG pathway enrichment analysis. Final data visualization was achieved using the R package ggplot2 [77].

### Cell cycle profile assay

Cells were collected and fixed in 70% pre-chilled ethanol and incubated overnight at –20 ℃. The cells were then washed with ice-cold 1X PBS and re-suspended in staining buffer (1X PBS containing 50 µg/mL of propidium iodide (#P3566, ThermoFisher), 100 µg/mL RNase A (Roche), and 0.1% of Triton X-100). The cells were incubated in the dark at room temperature for 30 min and then subjected to flow cytometry for cell cycle profile analysis (BD Biosciences). The results were analyzed using FCS Express 6 software (De Novo Software, USA).

### Senescence-associated β-galactosidase (SA-β-Gal) staining assay

The activity of β-galactosidase was determined by using an SA-β-Gal staining kit (#9860, Cell Signaling Technology) according to the manufacturer’s instructions. Briefly, control and LARS knockdown HepG2 cells were seeded into 6-well plates one day before the experiment. Cells were then incubated overnight with fresh staining solution in a 37 ℃ dry incubator (without CO_2_). Images were obtained using an EVOS XL Core Microscope (Life Technologies).

### Endogenous LC3B immunofluorescence (IF) staining

For immunofluorescence staining to detect endogenous LC3B protein, cells were cultured in an 8-chamber cell culture slide (CELLTREAT^®^) for 24 h and then fixed with ice-cold 100% methanol at –20 ℃ for 15 min. The cells were then permeabilized with 0.1% Triton X-100 solution at room temperature for 10 min and blocked with 3% BSA solution at room temperature for 1 h. Subsequently, cells were incubated overnight at 4 ℃ with anti-LC3B antibody (Cell Signaling Technology). The cells were washed three times with 1X PBS prior to incubation in 3% BSA solution containing 2.5 µg/mL DAPI (#D1306, ThermoFisher) and Alexa Fluor 488-conjugated secondary antibody (#R37118, Invitrogen) at room temperature for 1 h in the dark. After washing three times with 1X PBS and a final wash with distilled water, the cells were preserved in FluorSave^TM^ Reagent (#345789, Millipore) and imaged using a Zeiss LSM 700 Confocal Microscope at the Carl R. Woese Institute for Genomic Biology core facility (UIUC). The images were processed by ZEN (Zeiss) software and analyzed using ImageJ software.

### mCherry-GFP-LC3 transfection and confocal microscope

The tandem fluorescent-tagged LC3 reporter plasmid (mCherry–GFP–LC3; Addgene #110060) was used to monitor autophagic flux. Control and LARS knockdown HepG2 cells were seeded onto collagen-coated coverslips (#C3867, Sigma-Aldrich) in 6-well plates and transfected with the mCherry–GFP–LC3 plasmid using Lipofectamine 2000 (Invitrogen) according to the manufacturer’s instructions. At 48 h post-transfection, cells were treated with or without 25 nM bafilomycin A1 (Baf-A1; #B1793, Sigma-Aldrich) for 2 h, with DMSO used as the vehicle control. Cells were then washed with 1X PBS and fixed in 10% neutral buffered formalin for 30 min at room temperature, followed by permeabilization with 0.1% Triton X-100. Nuclei were counterstained with DAPI (2.5 µg/mL). Coverslips were mounted onto glass slides using FluorSave™ mounting medium (Millipore). Confocal images were acquired using a Zeiss LSM 700 microscope equipped with a Plan-Apochromat 63×/1.40 oil-immersion objective. The number of red puncta (GFP⁻/RFP⁺ LC3, autolysosomes) and yellow puncta (GFP⁺/RFP⁺ LC3, autophagosomes) per cell was quantified using ImageJ software.

### Transmission electron microscope (TEM)

Control and LARS knockdown HepG2 cells were harvested by trypsinization and washed twice with 1X PBS. The cells were then fixed in Karnovsky’s fixative (2% glutaraldehyde and 2.5% paraformaldehyde in 0.1 M phosphate buffer, pH 7.4) at 4 ℃ overnight. On the second day, cell samples were washed three times with Sorenson’s buffer and were then incubated in 2% (w/v) osmium tetroxide solution (#19170, Electron Microscopy Sciences) for 1 h at room temperature. Then 5% (w/v) potassium ferrocyanide solution (#25154-5, Electron Microscopy Sciences) was added to the samples and incubated for an additional 15 min. After extensive washes in water, samples were *en bloc* stained with saturated uranyl acetate (#4192, JT Baker Chemicals) in water and dehydrated in ascending concentrations of ethanol until the tissues were in 100% ethanol. Ethanol was replaced with pure acetonitrile, and samples were incubated for 10 min. Samples were then infiltrated with increasing concentrations of LX112 resin (#21210, Ladd Research) in acetonitrile mixtures. Finally, samples were embedded in 100% LX112 resin and polymerized at 60˚C overnight. Embedded samples were then cut into 70 nm sections using a diamond knife mounted on a Leica UC6 Ultramicrotome. Sections were collected on 200-mesh holey carbon-copper grids (#3620C-MB, SPI Supplies) and imaged using a JEOL JEM-1400 transmission electron microscope.

### Intracellular reactive oxygen species (ROS) measurement

Intracellular ROS levels were measured using the fluorogenic probe 2′,7′-dichlorofluorescein diacetate (H2DCF-DA; #287810, Sigma-Aldrich). Cells were incubated with 10 µM H2DCF-DA at 37 °C in the dark for 30 min, followed by three washes with 1X PBS. Fluorescence signals were captured using a Zeiss LSM 700 confocal microscope, and the fluorescence intensity was quantified by flow cytometry using a BD Biosciences flow cytometer.

### Mitochondrial membrane potential (MMP) evaluation

Mitochondrial membrane potential was assessed using the green fluorescent dye Rhodamine 123 (Rh123; #R302, Invitrogen). Cells were incubated with 2.5 µM Rh123 at 37 °C in the dark for 30 min, followed by three washes with 1X PBS. Fluorescence intensity was then analyzed by flow cytometry using a BD Biosciences flow cytometer.

### Nucleotide measurement by mass spectrometry

Control and LARS-knockdown cells were cultured to approximately 80% confluence. Cells were washed twice with ice-cold PBS, and intracellular metabolites were extracted using 80% methanol on ice. Following centrifugation to remove cellular debris, supernatants were collected, dried under vacuum, and reconstituted in acetonitrile (50:50, v/v) prior to analysis. For each sample, 1 μL of extract was injected for metabolomic analysis. Metabolite profiling was performed using an Agilent 1290 Infinity II UPLC system (Agilent Technologies, Santa Clara, CA, USA) coupled to an Agilent 6495 C triple quadrupole mass spectrometer. Data were acquired in both positive and negative electrospray ionization modes. Metabolite peak areas were integrated using Agilent MassHunter software. Metabolite abundances were normalized to cell mass and expressed relative to the corresponding control samples.

### Lysosome-mTOR co-localization analysis

Control and LARS-knockdown cells were cultured on collagen-coated glass coverslips. Cells were fixed with 4% paraformaldehyde, permeabilized with 0.2% Triton X-100, and blocked with 5% bovine serum albumin (BSA) in PBS. Cells were then incubated overnight at 4 °C with antibodies against LAMP1 (Alexa Fluor 488-conjugated; Santa Cruz Biotechnology, sc-20011 AF488) and mTOR (Cell Signaling Technology, #2983).

Following primary antibody incubation, mTOR staining was detected using an Alexa Fluor 568-conjugated goat anti-rabbit secondary antibody. Nuclei were counterstained with DAPI. Images were acquired using a Zeiss LSM 700 confocal microscope equipped with a Plan-Apochromat 63×/1.40 NA oil-immersion objective.

Co-localization between lysosomes (LAMP1) and mTOR was quantified by calculating Pearson’s correlation coefficient using ImageJ software. Multiple fields were analyzed for each experimental condition, and the average Pearson’s correlation coefficient was used to assess lysosomal localization of mTOR.

### Statistical analysis

Data are presented as mean ± standard deviation (SD) from three independent biological replicates unless otherwise indicated. Statistical analyses were performed using GraphPad Prism software. Comparisons among three groups (shControl, shLARS_1, and shLARS_2) were conducted using one-way analysis of variance (ANOVA) followed by Tukey’s multiple-comparisons test. For experiments involving only two groups, statistical significance was determined using a two-tailed unpaired Student’s *t* test. A *P* value < 0.05 was considered statistically significant. Statistical significance is indicated as follows: *P* < 0.05 (*)*, P* < 0.01 (**), *P* < 0.001 (***), and not significant (n.s.).

## Supplementary information

Supplementary figures

Supplementary figure legends

## Data Availability

All data used to support the findings in this study are presented in the paper and its supplementary materials. The datasets used and analyzed in this study are available from the corresponding author upon request.
