# Supplementary material for: LARS regulates cellular senescence and autophagy in liver cancer cells
Source: Cell Death Dis. 2026 Jul 17;17(1):885. doi: 10.1038/s41419-026-09114-0 (PMC13658823; doi:10.1038/s41419-026-09114-0)
Supplement: Supplementary file 1 [file 41419_2026_9114_MOESM2_ESM.docx]

**Supplementary Figure Legends:**

**Fig. S1.** Schematic illustration of the bioorthogonal noncanonical amino acid tagging (BONCAT) strategy used to assess nascent protein synthesis.

**Fig. S2. LARS knockdown does not induce EMT in HepG2 cells.** Immunoblot blot analysis of indicated EMT markers in control and LARS knockdown HepG2 cells. β-actin was used as a loading control.

**Fig. S3. LARS knockdown does not trigger apoptosis in HepG2 cells.** Immunoblot blot analysis of indicated proteins in control and LARS knockdown HepG2 cells. β-actin was used as a loading control.

**Fig. S4. LARS knockdown does not activate the DNA damage response (DDR) in HepG2 cells.** Immunoblot blot analysis of γ-H2AX in control and LARS knockdown HepG2 cells. Wild-type HepG2 cells treated with bleomycin at the indicated concentrations for 24 h were included as a positive control for DDR activation. β-actin was used as a loading control.

**Fig. S5. LARS knockdown activates p38 in HepG2 cells.** Immunoblot blot analysis of phosphorylated p38 and total p38 in control and LARS knockdown HepG2 cells. β-actin was used as a loading control.

**Fig. S6.** Relative levels of the indicated nucleotides in control cells and LARS-knockdown cells, measured by LC-MS.

**Fig. S7.** Immunoblot blot analysis of indicated proteins in control and LARS knockdown HepG2 cells. β-Tubulin was used as a loading control.
